# Evaluation of cardiac contractility of fetuses from pregestational diabetes mellitus pregnancies by three-dimensional ultrasound

**DOI:** 10.1590/1806-9282.20230700

**Published:** 2024-03-04

**Authors:** Zaqueu Caetano, Alberto Borges Peixoto, Nathalie Jeanne Bravo-Valenzuela, Rosiane Mattar, Edward Araujo

**Affiliations:** 1 Universidade Federal de São Paulo, Escola Paulista de Medicina, Department of Obstetrics – São Paulo (SP), Brazil.; 2 Universidade de Uberaba, Mário Palmério University Hospital, Gynecology and Obstetrics Service – Uberaba (MG), Brazil.; 3 Universidade Federal do Triângulo Mineiro, Department of Obstetrics and Gynecology – Uberaba (MG), Brazil.; 4 Universidade Federal do Rio de Janeiro, School of Medicine, Department of Pediatrics, Pediatric Cardiology – Rio de Janeiro (RJ), Brazil.

**Keywords:** Fetus, Heart contractility, Three-dimensional image, Diabetes mellitus

## Abstract

**OBJECTIVE:**

This study aimed to evaluate cardiac contractility in fetuses from pregestational diabetes mellitus pregnancies by three-dimensional ultrasound using spatiotemporal image correlation in rendering mode.

**METHODS:**

A retrospective cross-sectional study was performed on 40 fetuses from nondiabetic pregnancies and 28 pregestational diabetic pregnancies between 20 and 33 weeks and 6 days. Cardiac contractility was assessed by measuring the ventricular myocardial area in diastole subtracted from the ventricular myocardial area in systole.

**RESULTS:**

Pregestational diabetic pregnancies had a lower maternal age than nondiabetic pregnancies (26.7 vs. 39.9 years, p=0.019). Cardiac contractility in fetuses from diabetic and nondiabetic pregnancies was similar (p=0.293). A moderately positive and significant correlation was observed between gestational age and cardiac contractility (r=0.46, p=0.0004). A 1-week increase in gestational age was responsible for a 0.1386 cm^2^ increase in cardiac contractility.

**CONCLUSION:**

Cardiac contractility as evaluated by three-dimensional ultrasound using spatiotemporal image correlation in rendering mode showed no significant differences across fetuses with and without pregestational diabetes.

## INTRODUCTION

Assessment of fetal cardiac function is of great importance for monitoring conditions at high risk for heart failure, hydrops, and intrauterine death. Several techniques for determining fetal cardiac function by two-dimensional ultrasonography (2DUS) have been described, such as stroke volume, ejection fraction, cardiac output, shortening fraction, E/A ratio, and myocardial performance index^1^. Compared to fetuses from women with gestational diabetes mellitus, fetuses from pregestational diabetic pregnancies have significant left ventricular systolic and diastolic dysfunction^2^. Similarly, in comparison to normal-term fetuses and newborns, fetuses and newborns from diabetic women had cardiac indices indicative of myocardial compromise, reflecting a response to a relatively hyperglycemic intrauterine environment with altered fetal loading conditions and adaptation to subsequent acute changes in hemodynamic loading at delivery^3^.

Spatiotemporal image correlation (STIC) is the software available from the early 2000s on some three-dimensional ultrasound (3DUS) devices that capture a volume of the fetal heart, allowing off-line analysis in both static and dynamic multiplanar and rendering modes^4^. Rendering mode allows for the visualization of the interface of the cavities and walls of the heart, as well as for obtaining cardiac views not possible by 2DUS^5^. Moreover, it improves the understanding of anatomical defects in congenital heart disease (CHD) and communication between the multidisciplinary medical team and parents^6^. STIC in rendering mode has been used in evaluating measurements of various structures of the fetal heart such as interventricular septum area^7^ and ventricular myocardial area^8^.

The aim of this study was to evaluate the difference in myocardial area of the ventricles in diastole and systole in fetuses from pregestational diabetes mellitus pregnancies compared to fetuses from nondiabetic pregnancies by 3DUS with STIC in rendering mode, as a new method to evaluate fetal cardiac contractility.

## METHODS

This retrospective cross-sectional study was conducted at the Fetal Cardiology Sector, Discipline of Fetal Medicine, Department of Obstetrics, Paulista School of Medicine - Federal University of São Paulo (EPM-UNIFESP). Fetal heart volumes were selected in the database from 2012 to 2017. This study was approved by the Ethics Committee of UNIFESP.

Inclusion criteria were as follows: (1) singleton pregnancy; (2) gestational age between 20 and 33 weeks and 6 days; (3) absence of fetal cardiac malformations, as well as other congenital anomalies identified by ultrasonography; (4) electrical conduction disturbances of the heart; and (5) absence of chronic maternal diseases such as systemic arterial hypertension and collagenosis. Exclusion criteria were as follows: (1) conditions that would hinder and/or prevent adequate collection of fetal heart volumes such as abdominal scars or maternal body mass index ≥35 kg/m^2^ and (2) fetal spine between 11 and 1 o’clock. For the diagnosis of pregestational diabetes, the following criteria were used: HbA1c ≥6.5% (≥48 mmol/mol), random blood glucose ≥200 mg/dL (≥11.1 mmol/l), fasting blood glucose ≥126 mg/dL (≥7.0 mmol/dL), and 2-h oral glucose tolerance test ≥200 mg/dL (≥11.1 mmol/l).

All fetal cardiac volumes were collected in a Voluson E8 (General Electric, Healthcare, Zipf, Austria) device with a convex volumetric probe (RAB 4-8L), according to a technique standardized by Gonçalves et al^9^. For this, the four-chamber view was used as a reference, whenever possible in the ideal position (fetal spine positioned as close as possible to 6 h).

To acquire the volume of the region of interest (ROI), the entire fetal heart with its vascular connections was encompassed, in the smallest dimension possible. The settings for volume capture were opening angle between 20° and 40° and acquisition time between 10 and 15 s, which varied according to fetal movement. A single fetal heart volume was collected per pregnant woman, and all volumes were stored in the device and transferred to compact disks for later off-line reading.

During the study period, 4D fetal heart volume datasets were analyzed on a personal computer by a single examiner (ZC). All 4D fetal heart volume datasets were randomly selected. To perform the off-line analyses of fetal heart volumes, the examiner (ZC) used the program 4Dviews version 18.0 (General Electric, Healthcare, Zipf, Austria). Using this program, the fetal heart image was presented on the screen in multiplanar mode: axial or transverse (A), acquisition plane, sagittal or longitudinal (B), coronal or frontal (C), and magnified up to 1.23 times. Then, the axial plane (A) was selected, and the reference point was shifted to the crux cordis, and the image was rotated on the “z” axis so that the interventricular septum was arranged at 9 h. Then, the Render key was activated, with the following standardized post-processing: candle color, mixture of surface modes (80%) and surface smoothing (20%), threshold low (25), and transparency (50). The ROI selection key was selected, choosing the arrangement of the green line (ROI) in the forward/backward direction in the B (sagittal) plane. Then, the ROI line was displaced in the B plane (sagittal), forming a thick slice, as proposed by Gonçalves et al^9^.

**Figure 1.**
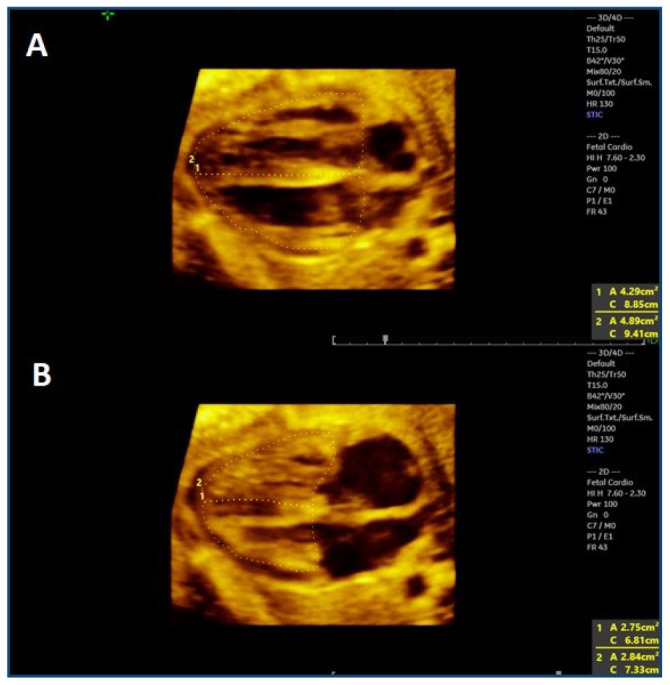
(A) Area measurement of the fetal ventricles in the rendering image of the four-chamber view in the plane of maximum filling. (B) Area measurement of the fetal ventricles in the rendering image of the four-chamber view in the plane of maximum closure.

Next, the 1×1 (full screen) layout was chosen, and by means of the cineloop, the exact view of maximum filling of the ventricles was obtained. The Measure key was selected, and the delimitation of the area by manual tracing was chosen. The area of both ventricles was delimited, with the interventricular septum divided in half. The same process was repeated after obtaining the plane of maximum closure of the ventricles (Figure 1). For the evaluation of cardiac contractility, the areas of the ventricles were subtracted from the maximum filling and closing of the ventricles.

Data were collected in an Excel 2007 spreadsheet (Microsoft Corp., Redmond, WA, USA) and analyzed using the SPSS statistical software version 20.0 (SPSS Inc., Chicago, IL, USA) and Prisma GraphPad version 7.0 (GraphPad Software; San Diego, CA, USA). The D’Agostino and Pearson normality test was used to analyze whether the values have a Gaussian distribution. Nonparametric distribution variables were presented as medians and minimum and maximum values. Parametric distribution variables were presented as the mean and standard deviation. To compare the variables between the groups, the Student’s t-test and Mann-Whitney test were used. The correlation between ventricular myocardial area during systole, ventricular myocardial area during diastole, difference of areas, and gestational age was performed using Pearson’s correlation test (parametric distribution variables) and Spearman’s correlation test (nonparametric distribution variables). Linear regression was performed to assess the ability of gestational age to predict ventricular myocardial area during systole, myocardial area during diastole, and the difference between the areas. The significance level (p)<0.05 was used in all analyses.

## RESULTS

A total of 81 pregnant women were evaluated, and 13 cases were excluded: 12 from nondiabetic pregnancies and 1 from pregestational diabetic pregnancy, due to technical difficulties in obtaining the measurements of the ventricular myocardial areas. Therefore, for the final statistical analysis, 68 fetuses were considered and divided into two groups: 40 fetuses from nondiabetic pregnancies and 28 fetuses from pregestational diabetic pregnancies, being 10 type II, 15 type I, and 3 Maturity-Onset Diabetes of the Young (MODY).

The clinical and echocardiographic characteristics of the study population are shown in Table 1. The demographic data (mean±standard deviation) of the diabetic group were as follows: fasting glucose 79.0±6.9 mg/dL (range, 50.0–91.0) and glycated hemoglobin 7.9±2.5% (range, 4.8–15.5). Pregestational diabetics had a lower maternal age than nondiabetic pregnancies (26.7 vs. 39.9 years, p=0.019, respectively). No significant difference was observed between the groups regarding gestational age at the time of ultrasound examination (p=0.620), ventricular myocardial area during systole (p=0.520), ventricular myocardial area during diastole (p=0.136), and difference between the areas (p=0.293).

**Table 1.** Clinical and echocardiographic characteristics of the study population.

	Normal (40)	Prior diabetes (28)	p
Maternal age (years)	30.9 (5.8)	26.7 (5.9)	0.019^†^
Gestational age (weeks)	27.2 (3.8)	26.7 (3.2)	0.620^†^
Myocardial area during ventricular systole (cm^2^)	4.42 (2.57)	4.04 (2.61)	0.520^†^
Myocardial area during ventricular diastole (cm^2^)	4.41 (2.57)	5.38 (2.61)	0.136^†^
Difference areas diastole – systole (cm^2^)	1.47 (0.26–5.37)	1.21 (0.10–3.59)	0.293^ƒ^

^†^Student’s t-test: Mean (standard deviation); ^ƒ^Mann-Whitney test: Median (minimum-maximum). p<0.05.

Considering all cases included in the study, there was a strong positive and significant correlation between gestational age and ventricular myocardial area during systole (r=0.62, p<0.0001). Although significant, 39% of ventricular myocardial area during systole was linearly related to gestational age. A 1-week increase in gestational age was responsible for increasing the area of myocardial contractility during systole by 0.4305 cm^2^.

Considering all cases included in the study, there was a strong positive and significant correlation between gestational age and ventricular myocardial area during diastole (r=0.64, p<0.0001). Although significant, 41% of ventricular myocardial area during diastole was linearly related to gestational age. Increasing gestational age by 1 week was responsible for increasing the area of ventricular myocardium during diastole by 0.5437 cm^2^.

Considering all cases included in the study, there was a moderately positive and significant correlation between gestational age and the difference in ventricular myocardial area during diastole and systole (r=0.46, p=0.0004). Although significant, 21% of the difference in ventricular myocardial area during diastole and systole was linearly related to gestational age. Increasing gestational age by 1 week was responsible for increasing the difference in ventricular myocardial area during systole and diastole by 0.1386 cm^2^.

## DISCUSSION

In this study, we sought to evaluate the ventricular myocardial contractility of fetuses from pregestational diabetic pregnancies by 3DUS using STIC in rendering mode. We did not observe any statistical differences between pregestational diabetic and nondiabetic pregnancies. This study has limitations such as retrospective design, exclusion due to technical difficulties, and a small number of cases. However, as a strength, to the best of our knowledge, this is the first study to propose a method to assess fetal cardiac contractility by 3DUS using STIC in rendering mode.

Experimental studies suggest that the hyperglycemic microenvironment during early embryogenesis can induce developmental heart defects by altering gene expression and cellular processes in the developing heart^10,11^. The hyperglycemic microenvironment within the uterus alters cardiac plasticity, characterized by electrical and structural remodeling of the heart^12^. In another experimental study, pregnancy in diabetic rats induced cardiac dysfunction, left ventricular hypertrophy, and an altered pro-inflammatory state in adult offspring only after a high-fat diet^13^.

In a recent study, 66 neonates from nondiabetic mothers, 60 neonates from well-controlled diabetic mothers, and 72 from poorly controlled diabetic mothers, tissue strain imaging showed a significant reduction in overall left ventricular strain and strain rate in the neonatal heart of poorly controlled diabetic mothers. The Tei index of left ventricular was higher in neonates of poorly controlled diabetic mothers compared to the other two groups. They concluded that neonates of diabetic mothers were at higher risk of developing cardiac abnormalities, including structural and functional defects^14^.

Three-dimensional ultrasound using the STIC and Virtual Organ Computer-Aided Analysis (VOCAL) methods has previously been used to assess cardiac function in normal fetuses^15^. Bravo-Valenzuela et al.^16^ compared 53 pregestational diabetic and 53 nondiabetic pregnancies between 20 and 34 weeks to assess fetal cardiac function by 3DUS using the STIC and VOCAL methods. The median left atrial volume in the diabetic group was significantly smaller than in normal pregnancies. Right and left ventricular volumes were similar in both groups. No significant differences were observed in ejection fraction, stroke volume, or cardiac output.

In this study, we chose to use the STIC in rendering mode, which consists of a virtual visualization of the fetal heart with all three dimensions combined. Lights and shadows are added to create a 3D effect. This virtual visualization illustrates the surface area of the heart with depth perception, making the image more realistic^17^. A previous study has proven the applicability and good intra- and interobserver reproducibility of STIC in rendering mode in assessing the myocardial area of the ventricles^8^. In a cross-sectional study, Melo Júnior et al.^18^ determined reference values for the total myocardial area by STIC in rendering mode in 168 fetuses between 20 and 33 weeks and 6 days. They observed a strong correlation of the area with gestational age, as well as good intra- and interobserver reproducibility. However, when compared to 30 fetuses from pregestational diabetic pregnancies, they observed no significant differences in area measurements.

In this study, we evaluated the difference in ventricular myocardial area in diastole and systole in order to determine a new parameter for contractility assessment by 3DUS using the STIC in rendering mode. We did not observe significant differences in myocardial contractility between normal fetuses and those from pregestational diabetic pregnancies. We believe that the lack of difference between the groups was due to the fact that the pregestational diabetic pregnancies were well controlled and all were on outpatient follow-up. Weber et al.^19^ evaluated 11 fetuses from nondiabetic and 9 fetuses from well-controlled insulin-dependent diabetic pregnancies who underwent serial assessments to determine cardiac growth and ventricular diastolic filling using M-mode and Doppler echocardiography during gestation and postpartum. They observed that good glycemic control in well-controlled insulin-dependent diabetic pregnancies resulted in normal cardiac growth and ventricular diastolic filling. Wong et al.^20^ evaluated eight well-controlled type I diabetes mellitus pregnancies with nine poorly controlled pregnancies at 30–32 weeks. They observed that there were no differences between the groups in fetal cardiac measurement, interventricular septal thickness measurement, ejection fraction, or peak systolic velocities of the pulmonary and aorta arteries. The E/A ratio of the tricuspid valve was significantly lower in poorly controlled diabetic pregnancies.

## CONCLUSION

We observed no significant differences in the cardiac contractility of fetuses from nondiabetic and pregestational diabetic pregnancies assessed by 3DUS using STIC in rendering mode. Accordingly, this technology has no additional advantages to be indicated in clinical practice for fetal cardiac contractility.
